# Potential molecular and cellular mechanisms for adverse placental outcomes in pregnancies complicated by SARS-CoV-2 infection—A scoping review

**DOI:** 10.1371/journal.pone.0283453

**Published:** 2023-03-23

**Authors:** Janelle Y. Wai, Eilidh M. Wood, Kylie K. Hornaday, Donna M. Slater

**Affiliations:** 1 Department of Physiology and Pharmacology, Cumming School of Medicine at the University of Calgary, Calgary, Canada; 2 Department of Obstetrics and Gynecology in the Cumming School of Medicine at the University of Calgary, Calgary, Canada; Virgen Macarena University Hospital, School of Medicine, University of Seville, SPAIN

## Abstract

**Background:**

Emerging evidence suggests that SARS-CoV-2 infection during pregnancy can result in placental damage and poor placental outcomes. However, the mechanisms by which SARS-CoV-2 infection leads to placental damage are not well understood. With a rapid expansion of literature on this topic, it is critical to assess the quality and synthesize the current state of literature. The objective of this scoping review is to highlight underlying mechanisms of SARS-CoV-2 mediated placental pathology in pregnant individuals and identify literature gaps regarding molecular and cellular mechanisms of poor placental outcomes.

**Methods:**

The review was conducted and reported following the recommendations of the PRISMA extension for Scoping Reviews. The study protocol was registered with Open Science Framework (https://osf.io/p563s/). Five databases (MEDLINE, EMBASE, Scopus, CINAHL, PubMed) were searched for studies published between September 2019 until April 2022. Studies assessing placental outcomes with respect to SARS-CoV-2 infection in pregnancy were eligible for inclusion. Outcomes of interest included histopathology, and molecular or cellular analysis. All records were uploaded into Covidence and extracted using the Joanna Briggs Institute method. Studies were assessed for risk of bias using the Newcastle Ottawa scale and a narrative synthesis of results was generated.

**Results:**

Twenty-seven studies reporting on molecular and/or cellular mechanisms of SARS-CoV-2 mediated placental outcomes were included in this review. SARS-CoV-2 infection was associated with perturbations in the ACE2 pathway, inflammatory mediators and immune cell populations and mitochondrial function in placentas.

**Conclusions:**

Our findings suggest that changes in the ACE2 pathway, mitochondrial dysfunction, and/or inflammatory processes may lead to placental damage observed in SARS-CoV-2 infection during pregnancy. More research is needed to understand the role of these pathways further, in addition to data collection related to trimester, severity, and strain.

## Introduction

In 2019, a novel coronavirus, severe acute respiratory syndrome coronavirus 2 (SARS-CoV-2), was discovered and made the headlines due to a rapid surge of infected individuals around the world. As of September 20^th^, 2022, there have been over 610 million confirmed cases and 6.5 million deaths attributed to SARS-CoV-2 reported to the World Health Organization [1]. The SARS-CoV-2 virus is an encapsulated single-stranded RNA virus which can cause relatively mild upper respiratory infections though disease outcomes may be worse in immunocompromised or vulnerable populations, such as pregnant women who undergo significant physiological and immunological changes through gestation [2].

During pregnancy, the placenta is a vital organ that mediates fetal oxygenation, nutrition, and protection from select pathogens. Thus, any changes to placental development and/or function may impact pregnancy outcomes and the health of the child (e.g., fetal growth restriction) and/or mother (e.g., pregnancy induced hypertension and/or preeclampsia) [3]. Emerging evidence shows SARS-CoV-2 infection of placental tissues is associated with syncytiotrophoblast infection and necrosis, along with aberrant infiltration of maternal macrophages into the intervillous space (histiocytic intervillositis) and increased perivillous fibrin deposition [4, 5]. Resultant placental damage likely leads to placental malperfusion and insufficiency, increasing risk of perinatal death via hypoxia, and potentially other adverse pregnancy outcomes [4, 6]. Severe cases of placentitis have been observed, including thrombohematoma, particularly in cases of still birth, and are likely a result of high placental viral load leading to severe placental damage [7]. However, the full impact of SARS-CoV-2 on placental function, physiology, and placental-related pregnancy outcomes, particularly the mechanism behind such processes, is not well understood [8–10].

Since the end of 2019, there has been a dramatic number of published papers related to SARS-CoV-2 with over 282,000 studies published under PubMed (November 2019-August 2022). However, with such a rapid expansion of novel studies, it is important to critically assess and synthesize the current state of the literature. Emerging literature assessing placental-related outcomes are often limited by small sample sizes with limited controls, vary in quality, and report contradictory results. Recent reviews have assessed SARS-CoV-2 infection with respect to neonatal and maternal pregnancy outcomes [11]. However, the emerging literature on placental-specific impacts of SARS-CoV-2 infection during pregnancy has yet to be reviewed. Specifically, we sought to review the evidence that highlights the underlying mechanism of SARS-CoV-2-mediated placental pathology and identify knowledge gaps with respect to molecular and/or cellular mechanisms of adverse placental outcomes. Thus, we conducted a scoping review to identify current evidence of placental outcomes with SARS-CoV-2 infection during pregnancy.

## Materials and methods

### Search strategy, timeline, sources

This study is a scoping review with the aim to identify key concepts and gaps in research pertaining to the effect of SARS-CoV-2 in pregnancy on the placenta and placental outcomes [12]. The review was conducted and reported by following the recommendations of the Preferred Reporting Items for Systematic reviews and Meta-Analyses extension for Scoping Reviews (PRISMA-ScR) [13]. The study protocol was registered with Open Science Framework (https://osf.io/p563s/). Five databases were searched for relevant literature: MEDLINE (Ovid), EMBASE (Ovid), SciVerse Scopus (Scopus), Cumulative Index to Nursing and Allied Health Literature (CINAHL), and the National Institutes of Health (PubMed). All databases were obtained through the University of Calgary Institution subscription. Databases were searched for records published between September 2019 until April 2022. Search terms included ‘placenta’ and ‘SARS-CoV-2’, as well as all relevant synonyms and alternate terms. Key terms were kept as similar as possible, including Medical Subject Headings (MeSH) terms for MEDLINE and adjusted syntax as appropriate (S1 File).

### Inclusion and exclusion criteria

Studies included primary observational studies related to placental outcomes with respect to SARS-CoV-2 diagnosis in pregnancy. Placental outcomes included those measured in primary placental tissue or cell samples, using histological, molecular, or biochemical analysis methods. Studies were excluded if they were animal studies, literature published before September 2019, randomized control trials and secondary studies (e.g. Systematic reviews, editorial reviews, commentaries, letters to editor, and expert opinion), case reports or abstracts with no quantifiable or experimental information, duplicated studies, reported study outcome not related to negative or positive placental outcomes (e.g. Histology, morphology, physiology, pathology) with respect to SARS-CoV-2 diagnosis during pregnancy, and papers published in a language other than English and without English translation (Table 1).

**Table 1:** Inclusion and exclusion criteria for data extraction.

Parameters	Inclusion Criteria	Exclusion Criteria
Study Population	Human studies	Animal studies
Study Duration	Literature published from September 2019 until April 2022	Literature published before September 2019 or after April 2022
Study Design	Primary studies including prospective study, retrospective study, cross sectional study, case studies, meta-analysis, observational studies	Randomized control trials, secondary studies including systematic reviews, editorial reviews, commentaries, letters to editor, expert opinion, and case reports or abstracts with no quantifiable or experimental information
Study Analysis	Any molecular or biochemical measurements assessing placentae (e.g., Stains, sequencing, Westerns, microscopy, mass spectrometry, gas chromatography, real time-PCR, qPCR, ELISA, aptamer-based proteomics)	Analysis methods using any molecular or biochemical measurements not assessing placentae
Study Outcome	Reported study outcome related to negative or positive placental outcome: molecular-physiology, cellular-physiology, histology, morphology, pathology, with respect to SARS-CoV-2 diagnosis	Reported study outcome not related to placental outcome: histology, morphology, physiology, pathology
Other	Sample collections of placentae *in vitro* and *ex vivo*	Published in a language other than English without English translation. Duplicated studies (Published papers at conference and later peer-reviewed, abstracts and later published study is peer-reviewed)—Only complete versions will be used

Abbreviations: PCR = polymerase chain reaction, qPCR = quantitative polymerase chain reaction, ELISA = enzyme-linked immunosorbent assay, SARS-CoV-2 = severe acute respiratory syndrome coronavirus 2.

Following the full text review, we noted that the included case reports and abstracts did not contain the required experimental information to contribute to an accurate assessment of placental outcomes and were therefore excluded from subsequent analysis.

### Data processing, quality assessment and analysis

Prior to literature screening all citations retrieved were exported into Covidence, a screening and data extraction software tool for creating literature reviews [14], and duplicates were removed. Title and abstracts were assessed for relevance, followed by the full text screening for final literature selection against the inclusion and exclusion criteria. All articles were independently assessed by three reviewers (JYW, DMS, EMW) and were included for data extraction if each paper received a ‘yes’ vote from two or more reviewers.

This review utilized a standardized data extraction template adapted from the Joanna Briggs Institute (JBI) Data Extraction Form designed for systematic reviews and research syntheses. Furthermore, the Newcastle Ottawa Scale was used to assess the quality of included cohort and case-control studies, as appropriate (S2 File).

Data extraction and risk of bias assessment were performed by three reviewers (JYW, KKH, EMW). Data was extracted and synthesized using MS Excel (S3 File).

### Outcomes

Included articles were grouped according to placental outcomes associated with SARS-CoV-2 infection. Group 1) histopathology, including morphological and histological assessment of placental morphology and function, such as by microscopy and/or histological and immunohistochemical assessment, Group 2) cellular and molecular analysis, including but not limited to gene expression, protein expression, cell/organelle physiology, and cell composition analysis of placentae. Though we will briefly review the literature in Group 1, the primary focus on this review is to critically assess and synthesize literature within Group 2, as these will highlight potential mechanisms of SARS-CoV-2-mediated adverse placental outcomes.

## Results

### Summary of search results

**Fig 1:**
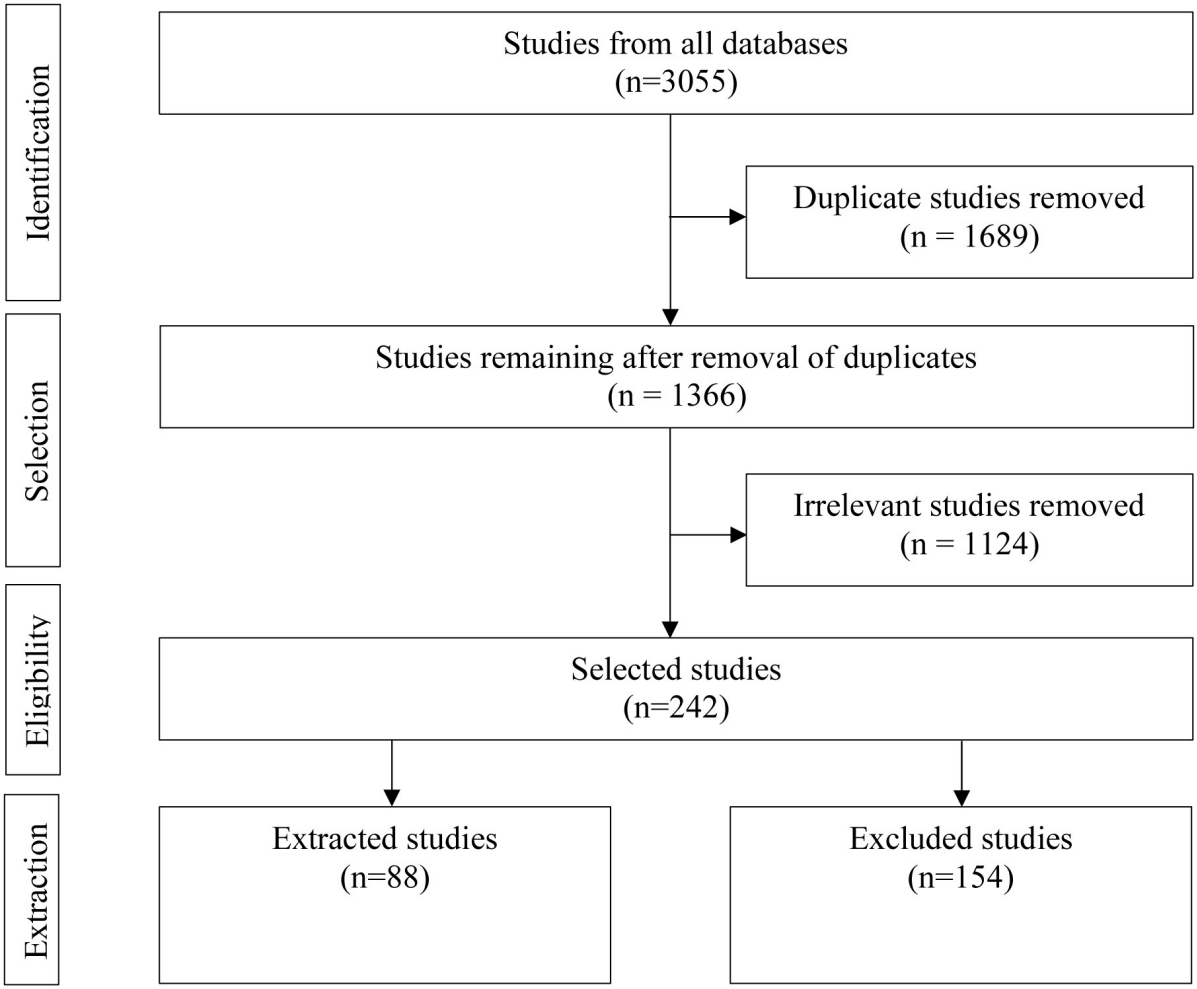
Flowchart using the Preferred Reporting Items for Systematic Reviews and Meta-Analyses extension for Scoping Reviews (PRSMA-ScR) for study selection.

The electronic search of the databases returned 1366 records after the duplicates (1689) were removed. Screening of the title and abstract resulted in the removal of 1124 as the studies were deemed irrelevant and not assessed further. A total of 88 studies met the inclusion criteria for review. During the initial title and abstract screening, three systematic reviews and/or meta-analyses [11, 15, 16] were identified with partial overlapping aims to the present review. The results in these reviews were limited to histopathological placental outcomes. To avoid redundancy, a full text analysis was not performed on those studies that strictly assessed histopathological outcomes in placenta. Instead, this review focused on those studies that assessed SARS-CoV-2-mediated molecular and/or cellular mechanisms of placental pathophysiology. Thus, the remaining 27 studies (Table 2), were included for full text review (Fig 1).

**Table 2:** Study characteristics and quality assessment of included studies.

Paper	Study Design	Country	Participants	Trimester With COVID-19	Severity	Quality Assessment
Argueta 2022 [19]	Cohort	USA	n = 2+ve	nr	nr	6/7
			n = 1-ve			
Benarroch 2021 [20]	Case-control	USA	n = 15+ve	3^rd^	nr	8/10
			n = 10-ve			
Bordt 2021 [21]	Case-control	USA	n = 38+ve	3^rd^ (median 38.7 weeks)	Asymptomatic (42%)	8/10
			n = 30-ve		Mild/ mod (50%)	
					Severe/ Critical (8%)	
Chen 2022 [22]	Case study	China	n = 1+ve	2^nd^	Severe (100%)	na
Cribiu 2021 [23]	Case-control	Italy	n = 21+ve	3^rd^	nr	7/10
			n = 16-ve			
Di Girolamo 2021 [11]	Systematic review and meta-analysis	USA, Italy, Switzerland, China, UK, Ireland, the Netherlands, Turkey, Belgium, Brazil, Spain, Mexico, France	n = 1008+ve	nr	Asymptomatic or symptomatic	na
Flores-Pliego 2021 [24]	Cohort	Mexico	n = 11+ve	nr	Mild (45.5%)	5/7
			n = 4-ve		Severe (54.5%)	
Gabanella 2022 [25]	Case Study	Italy	n = 2+ve	3^rd^	nr	na
Garcia-Flores 2022 [26]	Case-control	USA	n = 12+ve	nr	Asymptomatic (66.7%)	7/10
			n = 11-ve		Mild (8.3%)	
					Severe (25%)	
Hessami 2022 [15]	Systematic review and meta-analysis	USA, Italy, Spain, India, Brazil	n = 699+ve	3^rd^	Asymptomatic	na
			n = 18326-ve		Symptomatic	
Juttukonda 2022 [27]	Case-control	USA	n = 16+ve	2^nd^ or 3^rd^	Mild/ Mod (94%)	8/10
			n = 8-ve		Severe (6%)	
Lesseur 2022 [28]	Cohort	USA	n = 15+ve	nr	nr	6/7
			n = 30-ve			
Liu 2021 [29]	Cohort	China	n = 31+ve	nr	Mod (100%)	6/7
			n = 49-ve			
Mandò 2021 [30]	Cohort	Italy	n = 30+ve	3^rd^	Asymptomatic (40%)	5/7
			n = 16-ve		Symptomatic (60%) of which 16.7% Severe	
Mourad 2021 [31]	Case-control	USA	n = 66+ve	3^rd^	Asymptomatic/ mild (89.4%)	5/10
			n = 18-ve	(Asymp/ mild 38 weeks; severe 35.9 weeks)	Severe (10.6%)	
Nizyaeva 2021 [32]	Case-control	Russia	n = 66+ve	3^rd^	Asymptomatic (22.7%)	6/10
			n = 40-ve		Mild (38.0%)	
					Mod (30.2%)	
					Severe (9.1%)	
					Death (1.5%)	
Redline 2022 [33]	Case-control	USA	n = 271+ve	nr	Majority mild/ mod	7/10
			n = 8006-ve			
Rolfo 2022 [34]	Case-control	Italy	n = 41+ve	nr	Mild/ mod	9/10
			n = 12-ve			
Saulle 2021 [35]	Cohort	Italy	n = 15+ve	3^rd^	Asymptomatic	6/7
			n = 6-ve			
Shchegolev 2021 [36]	Case-control	Russia	n = 23+ve	nr	Mild/ mod	3/10
			n = 7-ve			
Sherer 2021 [37]	Case-control	USA	n = 22+ve	nr	Mild/ mod	6/10
			n = 11-ve			
Suhren 2022 [16]	Systematic review and meta-analysis	nr	n = 1452+ve	nr	nr	na
Sureshchandra 2021 [38]	Case-control	USA	n = 13+ve	1^st^ (7.7%)	Asymptomatic	4/10
			n = 19-ve	2^nd^ (15.4%)	Mild	
				3^rd^ (76.9%)		
Taglauer 2021 [39]	Case-control	USA	n = 16+ve	2^nd^ or 3^rd^	Mild/ mod	8/10
			n = 8-ve			
Verma 2021 [40]	Case-control	USA	n = 5+ve	2^nd^ (25%)	Asymptomatic or mild	6/10
			n = 5-ve	3^rd^ (75%)		
Wu 2021 [41]	Case-control	China	n = 11+ve	3^rd^ (22–31 weeks)	Mild/ mod	4/10
			n = 4-ve			
Zhao 2021 [42]	Cohort	China	n = 33+ve	nr	Mild/ mod	6/7
			n = 8-ve			

na = not applicable, nr = not reported, +ve = COVID-19 positive, -ve = COVID-19 negative, Mod = moderate.

### Summary of included studies

Selected studies included 15 case-control, 7 cohort, 2 case studies and 3 systematic reviews with meta-analysis. Studies included populations across 14 different countries, with the most common being the United States of America (14/27 studies). Most studies validated the presence of SARS-CoV-2 infection using PCR (polymerase chain reaction) based methods, and infection was most often detected in the third trimester, though only 15/27 studies described when in pregnancy infection was detected. Among studies that reported on severity of illness, most (16/21) used the terms asymptomatic, mild, moderate, and/or severe, although some only distinguished between asymptomatic and symptomatic illness. The majority of studies (11/21) did not report the criteria used to classify illness severity. Among those that did, two used published National Institutes of Health criteria [17], three used National Health Commission of the People’s Republic of China criteria [18], and five used a selected set of symptoms, with severe illness frequently being defined as illness requiring respiratory support. Only 4 / 27 studies reported on the variant of SARS CoV-2. Summarized in Table 2, see S3 File for full data extraction.

### Quality assessment

Overall, the methodological quality of cohort studies was good, with the most common score for l quality being 6/7, with a range of 5–7 of a total 7 possible points. For case-control studies, quality was generally good, with the most common score being 8/10, with a range between 3–10 points. The areas of lowest scoring for included papers were adequate controls, or comparability of cases and controls through matching/adjusting in analysis (S2 File). Out of the included studies, 5 were not applicable for quality assessments as they were meta-analyses or case studies. See Table 2 for details.

### Perturbations in pathways involved in placental vasculature

Two studies used immunofluorescence or immunohistochemistry to investigate proteins involved in endothelial structure. One found that von Wildebrand factor (vWf) levels, a clotting factor, increased in the decidual and chorionic villi endothelium with infection. Further, claudin-5, a tight junction protein involved in vascular permeability, and VE-cadherin (vascular endothelial cadherin), a major determinant of endothelial contact that also determines vascular permeability, decreased with infection. These patterns were stronger in cases of severe infection compared to mild [24]. The other found that vascular endothelial growth factor, (VEGF) an angiogenic inducing factor, increased with infection [36]. See S3 File for data extraction and details of studies.

### Changes in pathways involved in SARS-CoV-2 viral entry

Several papers reported differences in expression of genes and or levels of proteins involved in the angiotensin converting enzyme two (ACE2) pathway. In placentas from participants with SARS-CoV-2 infection during the third trimester, expression of ACE2, protein and mRNA, were reduced compared to controls [39, 40]. In placenta from participants with infection during the second trimester this pattern was not observed [39]. ACE2 levels were also reported to be lower in asymptomatic/mild infections compared to severe infection [31]. Infection was reported to be associated with increased levels of a disintegrin and metalloprotease domain 17 (ADAM17) [39]. No differences were found in expression levels of transmembrane protease serine 2 (TMPRSS2) [31] in infected compared to SARS-CoV-2 negative controls. Infection was reported to be associated with decreased levels of the protease furin in one study [28] while another found no differences in infected compared to SARS-CoV-2 negative controls [25].

Additionally, infection was associated with changes in proteins involved in endocytosis including increased levels of ras-related in brain 5 (RAB5) and decreased levels of ras-related in brain 7 (RAB7) [20]. Infection was also associated with upregulation of miRNAs that act on viral replication [35].

### Placental mitochondrial function

Levels of thiobarbituric acid (TBARS) (lipid peroxidation marker), hypoxia inducible factor 1 alpha (HIF-1a), catalase (CAT), superoxide dismutase (SOD), and glutathione synthetase (GSS) are higher in SARS-CoV-2 positive placentas, indicating perturbations in hypoxic response and mitochondrial physiology [30, 34]. Respiratory chain subunit genes NADH:Ubiquinone oxidoreductase subunit A9 (NDUFA9), succinate dehydrogenase complex flavoprotein subunit A (SDHA) and cyctochrome C oxidase subunit 4I1 (COX4I1) were also decreased in symptomatic infected cases [30]. Mitochondrial DNA (mtDNA) was also decreased in infected placentae, potentially due to increased DNA oxidative damage [30], though another study found elevated mtDNA in infected placentae [29], indicating that further research is necessary to tease out the effect of SARS-CoV-2 infection on mtDNA levels. SARS-CoV-2 infection was also associated with aberrant mitochondrial reticulum and an increase in mitochondrial fission over fusion, accompanied by a decrease in genes involved in mitochondrial dynamics dynamin-1-like protein (DNM1L) and mitochondrial fission 1 protein (FIS1) [30] resulting in highly fragmented mitochondria [25].

### Immune cells and inflammatory mediators measured in placenta

SARS-CoV-2 infection is associated with perturbations in placental immune cell infiltration [38], including increased subchorionic infiltration of neutrophils [40], intervillous infiltration of maternal macrophages and T-cells [19], elevated cluster of differentiation 14 (CD14+) macrophages [41], third trimester decidual infiltration of macrophages and natural killer (NK) cells and second trimester increases in T-cells [27]. However, the neutrophil to macrophage ratio is much higher in early, localized placentitis as compared to diffuse infection [33]. Infection is also associated with increased levels of inflammatory cytokines including interferon stimulated genes (IFI6, OAS1, CCL3) and proteins (IFNa, IFNy, CCL4), interleukin-10 (IL-10) expression [21], CXC-motif chemokine ligand 10 (CXCL10) [18, 25], tumor necrosis factor alpha (TNFa), CXC-motif chemokine ligand 8 (CXCL8) [32], interferon gamma-induced protein 10 (IP-10), interferon gamma/ monokine induced by interferon-gamma IFNy/MIG [41], C-C motif chemokine ligand 3 and 5 (CCL3, CCL5), interleukin-6 (IL-6), interleukin-1-beta (IL1B), interferon alpha (IFNa), and interferon beta (IFNB) [35] and a decrease in interleukin-4 (IL-4) and macrophage migration inhibitory factor (MIF) expression [32]. Infection was also associated with an increase in host antiviral effector gene expression including MX dynamin like GTPase 1 (MX1), interferon induced transmembrane protein 1 and 3 (IFITM1, IFITM3), cholesterol 25-hydroxylase (CH25H), toll-like receptor 3 (TLR3), and DExD/ H-box helicase 58 (DDX58) [35], and increased expression of genes and pathways involved in inflammatory response to viral infection [19]. However, other studies found no significant differences in IL1B, IL6, TNFa, or CXCL8 mRNA levels with infection [37], and that pro-inflammatory cytokines (IL-6, CXCL8, IL-10 and TNFa) were downregulated with infection [27].

Single cell RNA sequencing (scRNAseq) analysis demonstrated activation of CD8 T-cells and overactivation of macrophages [22]. Increased CD68+ macrophage infiltration was found in the placenta even after recovery from SARS-CoV-2 infection [42]. Another scRNAseq investigation revealed differential expression primarily within the maternal T cells and macrophages in the chorioallantois membrane as well as decidual and lymphatic endothelial cells of maternal origin [26]. In contrast, one study found no change in gene expression in n = 21 infected placentae compared to healthy placentae, except for one placenta with severe viral load, in which they observed high expression of genes implicated in innate antiviral immunity, chemotactic and inflammatory response and adaptive response [23].

### Other gene expression in placenta

Single cell RNA sequencing (scRNAseq) analysis demonstrated impaired trophoblast differentiation and lowered expression of syncytiotrophoblast related genes [19]. SARS-CoV-2 infection was associated with decreased levels of trophoblast cell type markers pregnancy specific beta-1-glycoprotein 3 (PSG3), and chorionic gonadotropin subunit beta 3 (CGB3) [28].

See S3 File for full data extraction and details related to findings in the placenta.

## Discussion

### Summary of main findings

There is an emerging and rapidly expanding field of literature demonstrating that SARS-CoV-2 infection during pregnancy is associated with placental damage, as evidenced by histopathological assessment of infected placentas (recently reviewed in [11, 15, 16]). However, there continues to be a significant gap in knowledge surrounding the molecular underpinnings of this SARS-Co-V-2 related placental damage. Based on the papers identified for the current scoping review, preliminary evidence points to changes in differential gene expression in key pathways such as renin angiotensin system (RAS) pathway, mitochondrial dysfunction, and inflammatory processes. Yet, how these pathways are linked to placental lesions and subsequent adverse maternal and neonatal outcomes remains unclear.

### SARS-CoV-2 infection during pregnancy leads to poor placental outcomes

SARS-CoV-2 infection during pregnancy has been found to be associated with placental abnormalities, including maternal vascular malperfusion (MVM), fetal vascular malperfusion (FVM), and increased perivilous fibrin deposition, as recently reviewed [11, 15, 16]. Placental changes suggest hypoperfusion of the vasculature and inflammation of the placental tissue, as well as perturbations in endothelial vascular structure [24], at least in most placentas with symptomatic infection. Hypoperfusion of the placenta may be involved in the mechanism of pre-eclampsia and may lead to fetal growth restriction because of poor blood flow [43]. Complications reported to be associated with SARS-CoV-2 infection include decreased fetal movement [44], pregnancy loss [45], caesarean section [46], preterm delivery [33, 47, 48], hypertension [48], and preeclampsia [10, 47]. In contrast, some studies show that outcomes are not different in those pregnancies complicated by SARS-CoV-2, including rates of stillbirth [49], PTB or preeclampsia [50, 51], or caesarean section for fetal indication [50]. However, further studies would be required to elucidate the link between placental lesions and pregnancy outcomes with respect to SARS-CoV-2 infection.

### Potential mechanism of placental dysfunction

Review of the evidence revealed significant perturbations in expression of genes involved in the ACE2 and RAS genes in placentae from pregnancies complicated by SARS-CoV-2 infection. The virus enters cells by binding its main receptor ACE2 and enters through two proposed mechanisms either via plasma membrane fusion, which is dependent on the cleavage of the viral spike (S) protein by TMPRSS2, or via endocytosis, in which the S protein is cleaved inside the cell by other proteases such as furin [52]. There is conflicting evidence on whether TMPRSS2 and ACE2 are co-localized in the placenta, and thus the primary mode of viral entry in placental cells remains unclear [53, 54]. ACE2 plays an important role in balancing the RAS, which is a critical regulator of blood volume and vascular resistance. Briefly, renin acts to produce AngI from its precursinteror angiotensinogen, AngI can be subsequently converted to AngII by ACE. AngII binds primarily to the angiotensin II receptor 1 (AT1) to promote vasoconstriction. ACE2 plays a counter-regulatory role by converting AngII to Ang1-7, which binds to the G-protein coupled receptor (GPCR) Mas to promote vasodilation [55]. Depletion of ACE2 levels due to sequestration by SARS-CoV-2 may lead to associated effects on the RAS system which in turn may be linked to placental pathology and associated adverse pregnancy outcomes [39, 40, 56].

Levels of ACE, ACE2 and Ang1-7 are all significantly increased during pregnancy [57], which suggests that RAS plays an important role in maternal hemodynamics, such as to facilitate increased blood volume and flow to support the growing fetus. Soluble ACE2, or sACE2, is secreted from the plasma membrane through shedding from the plasma membrane, stimulated by the metalloproteinase ADAM17 [58]. Soluble ACE2 may contribute to host defense against SARS-CoV-2 as sACE2 can bind free virions, sequestering them from entering cells, though ACE2 levels are typically ~1000x higher than sACE2 [59]. High levels of ADAM17 and increased activity in placental villous tissue may indicate that ACE2 shedding is an important response to infection in the placenta and upregulation of ADAM17 may be protective against SARS-CoV-2 related placental outcomes [39].

Placentation, which occurs primarily in the first 10–14 weeks of gestation, is regulated by the RAS pathway, indicating that SARS-CoV-2 infections, primarily those that occur in the first trimester, may impact proper placentation and placental function. AngII has been described to stimulate the migration and invasion of extravillous trophoblast during placentation, and AngII and AT1 levels are higher at weeks 10–14 compared to term [60, 61]. AngII/AT1 action can also stimulate vascular endothelial growth factor (VEGF) secretion to promote angiogenesis [62]. Further, an ACE insertion/deletion polymorphism, which effects ACE expression and levels, is a common risk factor for pre-eclampsia, preterm birth, and recurrent pregnancy loss [52]. Insufficient invasion and placental hypoperfusion, potentially due to insensitivity to or low levels of AngII, is linked to poor maternal and fetal outcomes such as pre-eclampsia and fetal growth restriction [63, 64]. Conversely, excessive trophoblast invasion can lead to placenta accreta, a condition characterized by placental growth deep into the myometrial wall, which is a serious condition that can lead to postpartum hemorrhage. The ACE2/Ang1-7/Mas axis acts as a counter-regulator of inflammation, proliferation, and angiogenesis and thus may play an important role in fine-tuning placentation and placental function.

ACE2 overexpression has been shown to stimulate the expression of mitochondrial related genes, indicating that ACE may also play an important role in mitochondrial function [65]. Perturbations in the hypoxic response and mitochondrial physiology may contribute to abnormal placental function and adverse pregnancy outcomes such as preeclampsia, which is characterized in part by placental ischemia [66]. Excess mitochondrial fission is linked to ischemia in a rat model of cardiac dysfunction [67], and mitochondrial homeostasis is critical for hypoxic response in placenta [68]. Mitochondrial dysfunction may also contribute to oxidative damage in placental tissues, which in turn is linked to several adverse pregnancy outcomes including preeclampsia and intrauterine growth restriction [69]. Though there is evidence to suggest perturbations to mitochondrial physiology of the placenta during SARS-CoV-2 infection, further research would be required to determine how viral infection impacts mitochondrial physiology, and the mechanism by which this leads to adverse pregnancy outcomes.

SARS-CoV-2 infection was also found to be associated with significant perturbations to immune cell populations and inflammatory mediators. It is currently unclear whether perturbations to immune and inflammatory mediators during SARS-CoV-2 infection contribute to placental pathology or are a response to ongoing infection. NK cells and macrophages are prominent in the placental bed during placentation and not only play important roles in immune regulation and innate response during pregnancy, but also play a role in regulation of angiogenesis, trophoblast invasion, and spiral artery remodeling [70]. Acute and chronic inflammation of the placenta, such as in the case of infection, is also linked to long term developmental outcomes [71], though future research is required to tease out the effect of SARS-CoV-2 on immune- and inflammatory-mediated placental function.

## Considerations for future research

As common in newly emerging diseases, case series studies were frequent among the literature reviewed. As such, these studies often had no controls or the controls were not comparable, such as hospitalized and/or nonpregnant controls. Future studies with clearly defined pregnant, uninfected healthy controls would be necessary to tease out SARS-CoV-2 specific placental outcomes. SARS-CoV-2 positive cases included in this review were disproportionately diagnosed with infection in their third trimester. This is likely due to when participants are most likely to receive testing, such as through routine hospital screening at presentation for delivery. Given the contradictions in the literature to date, in that some authors suggest timing of infection does not impact placental pathology [28, 72], while others report the risk of developing some placental pathologies, particularly FVM, differs based on timing of infection [73], further research in regard to gestational age and infection is required. In addition, further research is needed to investigate whether SARS-CoV-2 infections earlier in pregnancy have a greater impact on placentation and RAS-mediated trophoblast invasion.

The severity of symptoms is also an important consideration. For example, some studies suggest that increased disease severity corresponds to a higher instance of placental lesions and malperfusion [36], other studies suggest that neither timing nor severity of infection impact placental pathology [28, 47]. As ACE2 depletion was greater in asymptomatic/mild infections compared to severe [31], it is possible that RAS dysregulation may vary based on viral load. Due to lack of available genotyping data in the current search, we were unable to comment on the effect of SARS-CoV-2 variant on the prevalence and severity of placental pathology, morphology, and physiology, though it has been suggested that mutations to the S protein may increase transmissibility and pose a greater threat to both the pregnant and unborn population [19]. Further research with defined controls and clear case groups with information on severity and timing of infection is critical for teasing out the complexity of SARS-CoV-2 infection in pregnancy.

This review also identified a significant gap in knowledge surrounding heterogeneity in SARS-CoV-2 mediated placental outcomes. It is widely accepted that SARS-CoV-2 outcomes differ based on socioeconomic status (SES), age, Indigenous and other ethnic status, comorbidities, and barriers to healthcare access [74]. However, there is limited pregnancy- and placenta-specific research on how SARS-CoV-2 may impact different individuals. Many kinds of social determinants of health can also impact pregnancy outcomes adversely. For example, advanced maternal age, higher BMI, ethnicity such as African descent, and lower SES have all been previously linked to poor pregnancy outcomes leading to preterm birth and preeclampsia [75], though it is unclear whether this would be further complicated by SARS-CoV-2 comorbidity.

### Strengths and limitations

This scoping review abides by the PRISMA-ScR reporting guidelines and was preregistered to promote transparency in the review process. In the process of the current review, we identified a previous study reviewing histopathological effects of SARS-CoV-2 on placentae. However, this is the first review, to our knowledge, that synthesizes the available literature which highlights molecular and cellular studies and the potential mechanisms of placental damage with SARS-CoV-2 infection during pregnancy.

The current review did not include grey or unpublished literature. As a rapidly emerging field, SARS-CoV-2 related research is continuously evolving, and the authors are hopeful that further strides will be made in elucidating the mechanism of placental dysfunction during SARS-CoV-2 infection. Further, due to limitations in resources, we were unable to include those studies written in a language other than English. Though our review did include studies from 25 different countries worldwide, there is likely a bias towards English-speaking populations or those areas where English translations would be common.

## Conclusion

This scoping review synthesizes the literature regarding placental pathology, histology, morphology, and physiology to assess the present state of literature and identify knowledge gaps for future research. While it is largely established that SARS-CoV-2 causes placental damage, the underlying mechanisms that explain why this occurs is not clear. In this review we highlight evidence that suggests a role for the immune system, altered gene expression, or mitochondrial dysfunction in the process. Further investigations into the potential role of these molecular and cellular pathways are required, in addition to collecting more data related to trimester, severity, and SARS-CoV-2 strain of infectivity.

## Supporting information

S1 File Search strategy. (DOCX)

S2 File Methodological quality assessment data. (DOCX)

S3 File Data extraction. (DOCX)

S4 File Acronyms and abbreviations. (DOCX)

S1 Checklist PRISMA ScR checklist. (DOCX)

S2 Checklist PRISMA abstract checklist. (DOCX)
