# Supplementary material for: Potential molecular and cellular mechanisms for adverse placental outcomes in pregnancies complicated by SARS-CoV-2 infection—A scoping review
Source: PLoS One. 2023 Mar 23;18(3):e0283453. doi: 10.1371/journal.pone.0283453 (PMC10035918; doi:10.1371/journal.pone.0283453)
Supplement: S1 File — (DOCX) [file pone.0283453.s001.docx]

**ST1 File. Search strategy for databases.**

MEDLINE and EMBASE search strategy

| **Concept** | **MeSH Term** | **Other terms** |
| --- | --- | --- |
| Placenta | Placenta | Placent*  Uterus  Fetal Membrane |
| COVID-19 | COVID-19  Coronavirus | nCoV*  2019nCoV  19nCoV  COVID19*  COVID  SARS-COV-2  SARSCOV-2  SARS-COV2  SARSCOV2  SARS coronavirus 2  Severe acute respiratory syndrome coronavirus 2 Severe acute respiratory syndrome corona virus 2  (Coronavirus* or betacoronavirus or coronavirus infections) and (disease outbreaks or epidemic or pandemic) |

Search strategy:

(Placent* or Uterus or Fetal Membrane).tw.kf.

exp Placenta/

1 or 2

(Coronavirus* or betacoronavirus or coronavirus infections) and (disease outbreaks or epidemic or pandemic)

(nCoV* or 2019nCoV or 19nCoV or COVID19* or COVID or SARS-COV-2 or SARSCOV-2 or SARS-COV2 or SARSCOV2 or SARS coronavirus 2 or Severe acute respiratory syndrome coronavirus 2 or Severe acute respiratory syndrome corona virus 2).ti.ab.kf.

exp COVID-19/

exp Coronavirus/

4 or 5 or 6 or 7

3 and 8

Limit 9 yr=”2019 -Current”

CINAHL search strategy

| **Concept** | **Field** | **Other terms** |
| --- | --- | --- |
| Placenta | Title  Abstract  Heading | Placent*  Uterus  Fetal membrane |
| Coronavirus | Title  Abstract  Heading | (Coronavirus* or betacoronavirus or coronavirus infections) and (disease outbreaks or epidemic or pandemic) |
| COVID-19 | Title  Abstract  Heading | nCoV*  2019nCoV  19nCoV  COVID19*  COVID  SARS-COV-2  SARSCOV-2  SARS-COV2  SARSCOV2  SARS coronavirus 2  Severe acute respiratory syndrome coronavirus 2 Severe acute respiratory syndrome corona virus 2 |

Search strategy:

( (TI Placent* OR AB Placent*) ) OR ( (TI Uterus OR AB Uterus) ) OR ( (TI Fetal membrane OR AB Fetal membrane) ) )

(MH “Placenta+”)

S1 OR S2

TI ( (Coronavirus* or betacoronavirus or coronavirus infections) and (disease outbreaks or epidemic or pandemic) ) OR AB ( (Coronavirus* or betacoronavirus or coronavirus infections) and (disease outbreaks or epidemic or pandemic)

TI ( (nCoV* or 2019nCoV or 19nCoV or COVID19* or COVID or SARS-COV-2 or SARSCOV-2 or SARS-COV2 or SARSCOV2 or SARS coronavirus 2 or Severe acute respiratory syndrome coronavirus 2 or Severe acute respiratory syndrome corona virus 2) ) OR AB ( (nCoV* or 2019nCoV or 19nCoV or COVID19* or COVID or SARS-COV-2 or SARSCOV-2 or SARS-COV2 or SARSCOV2 or SARS coronavirus 2 or Severe acute respiratory syndrome coronavirus 2 or Severe acute respiratory syndrome corona virus 2) )

(MH “COVID-19”)

(MH “Coronavirus+”)

(S1 OR S2) AND (S3 OR S4 OR S5 OR S6)

Limiters – Published Date: 20190901-20220431

Scopus search strategy

| **Concept** | **Field** | **Other terms** |
| --- | --- | --- |
| Placenta | Title-abs-key | Placent*  Uterus  Fetal membrane |
| Coronavirus | Title-abs-key | (Coronavirus* or betacoronavirus or coronavirus infections) and (disease outbreaks or epidemic or pandemic) |
| COVID-19 | Title-abs-key | nCoV*  2019nCoV  19nCoV  COVID19*  COVID  SARS-COV-2  SARSCOV-2  SARS-COV2  SARSCOV2  SARS coronavirus 2  Severe acute respiratory syndrome coronavirus 2 Severe acute respiratory syndrome corona virus 2 |

Search strategy:

( TITLE-ABS-KEY ( ( ( placent* OR uterus OR ( fetal AND membrane ) ) ) )

AND

TITLE-ABS-KEY ( ( ( ( coronavirus* ) OR ( betacoronavirus ) OR (coronavirus AND infections ) ) AND ( ( disease AND outbreaks ) OR ( epidemic ) OR ( pandemic ) ) ) )

OR

TITLE-ABS-KEY ( ( ( ncov* ) OR ( 2019ncov ) OR ( 19ncov OR covid19 ) OR ( covid ) OR ( sars-cov-2 ) OR ( sarscov-2 ) OR ( sars-cov2 ) OR ( sarscov2 ) OR ( sars AND coronavirus AND 2 ) OR ( severe AND acute AND respiratory AND syndrome AND coronavirus AND 2 ) OR ( severe AND acute AND respiratory AND syndrome AND corona AND virus AND 2 ) ) ) )

AND

PUBYEAR > 2018

PubMed search strategy

| **Concept** | **Field** | **Other terms** |
| --- | --- | --- |
| Placenta | Title  Abstract  Heading | Placent*  Uterus  Fetal membrane |
| Coronavirus | Title  Abstract  Heading | (Coronavirus* or betacoronavirus or coronavirus infections) and (disease outbreaks or epidemic or pandemic) |
| COVID-19 | Title  Abstract  Heading | nCoV*  2019nCoV  19nCoV  COVID19*  COVID  SARS-COV-2  SARSCOV-2  SARS-COV2  SARSCOV2  SARS coronavirus 2  Severe acute respiratory syndrome coronavirus 2 Severe acute respiratory syndrome corona virus 2 |

Search strategy:

((Placent*) OR (Uterus)) OR (Fetal Membrane)

COVID-19 [mh] OR ((Coronavirus[mh:noexp]
OR Betacoronavirus[mh:noexp] OR Coronavirus Infections[mh:noexp]) AND (Disease Outbreaks[mh:noexp] OR Epidemics[mh:noexp] OR Pandemics[mh])) OR nCoV[tiab] OR nCoV[tt] OR 2019nCoV[tiab] OR 2019nCoV[tt] OR 19nCoV[tiab] OR COVID19*[tiab] OR COVID19*[tt] OR COVID[tiab] OR COVID[tt] OR SARS-CoV-2[tiab] OR SARS-CoV-2[tt] OR SARSCOV-2[tiab] OR SARSCOV-2[tt] OR SARS-COV2[tiab] OR SARS-COV2[tt] OR SARSCOV2[tiab] OR SARSCOV2[tt] OR SARS coronavirus 2[tiab] OR SARS coronavirus 2[tt] OR Severe Acute Respiratory Syndrome Coronavirus 2[tiab] OR Severe Acute Respiratory Syndrome Coronavirus 2[tt]

(#1) AND (#2)

Filters: from 2019/9/1 – 2022/4/30
