# Supplementary material for: Potential molecular and cellular mechanisms for adverse placental outcomes in pregnancies complicated by SARS-CoV-2 infection—A scoping review
Source: PLoS One. 2023 Mar 23;18(3):e0283453. doi: 10.1371/journal.pone.0283453 (PMC10035918; doi:10.1371/journal.pone.0283453)
Supplement: S2 File — (DOCX) [file pone.0283453.s002.docx]

**ST2 File. Methodological quality assessment rubrics**

Methodological quality assessment rubric for case control studies

|  | **2pt** | **1pt** | **0pt** |
| --- | --- | --- | --- |
| **Is the case definition adequate?** | Subjects have a confirmed positive covid diagnosis during pregnancy, (first, second or third trimesters) with molecular results with respect to positive SARS-CoV-2 diagnosis. independently validated by primary records available including PCR test and/or HCP supervised antigen testing | Molecular results with respect to positive SARS-CoV-2 diagnosis. Self-reported or record linkage with no reference to primary record | No description of case/control definition (studies which did not include subjects with covid during pregnancy eg. Covid before or after pregnancy, were not included) |
| **Representativeness of the cases** |  | Study includes consecutive or obviously representative series of cases: all cases with outcome of interest over a defined period of time, all cases in a defined catchment area, all cases in a defined hospital or clinic, group of hospitals, health maintenance organization, or an appropriate sample of those cases (e.g. random sample) | Potential for selection biases (did not meet requirements in cell D3) or not stated |
| **Selection of controls** |  | Controls used in the study is derived from the same population as the cases and essentially would have been cases had the outcome been present (same community) | No description, controls not from same community |
| **Definition of controls** |  | If cases are first occurrence of outcome (i.e. no history of COVID-19), then it must be explicitly stated that controls have no history of covid-19. If cases have new (not necessarily first) occurrence of covid-19, then controls with previous occurrences of covid-19 should not be excluded | No mention of history of covid-19 |
| **Comparability of cases and controls on the basis of the design or analysis** |  | Cases and controls are matched in the design and/or confounders are adjusted for in the analysis | Cases and controls are not matched and/or confounders are not adjusted for in the analysis. Comparability of cases and controls show evidence of bias. |
| **Ascertainment of exposure** | Placental outcomes related to histology, morphology, pathology, physiology are measured using methods that are sufficiently described so as to be repeatable, researcher blinded to case/control status | Methods insufficiently described so as to be repeatable, researcher not blinded to case/control status | no description (insufficient) |
| **Same method of ascertainment for cases and controls** |  | Placental outcomes were measured using identical methods for cases and controls | Placental outcomes were measured using different methods in cases and controls |

Adapted from the Newcastle Ottawa Scale

Methodological quality assessment rubric for cohort studies

|  | 2pt | 1pt | 0pt |
| --- | --- | --- | --- |
| **Representativeness of the cohort** | Truly or somewhat representative of the average pregnant community | selected group (e.g. volunteers) | no description of the derivation of the cohort |
| **Ascertainment of exposure** | Positive or negative placental findings related to histology, morphology, pathology, physiology, and molecular results with respect to positive SARS-CoV-2 diagnosis, with test methods/ results sufficiently described so as to be repeatable | Positive or negative placental findings related to histology, morphology, pathology, physiology, and molecular results with respect to positive SARS-CoV-2 diagnosis, with test methods/ results insufficiently described so as to be repeatable | No description (insufficient) |
| **Assessment of outcome** | Positive SARS-CoV-2 infection in pregnant women during their pregnancy, independently validated with reference to primary medical/hospital records | Positive SARS-CoV-2 infection in pregnant women during their pregnancy, self-reported or record linkage with no reference to primary record | No description (*note: studies which did not describe outcome as positive or negative SARS-CoVCoV-2 infection were excluded) |
| **Adequacy of follow up of cohorts** |  | Complete follow-up, all subjects accounted for, or subjects lost to follow up unlikely to introduce bias (small number or a description provided of those lost) | Subjects lost presents likely introduction of bias and/or no description provided for those lost |

Adapted from the Newcastle Ottawa Scale

Methodological quality assessment of included case control studies

| **Study** | **Case Definition** | **Representativeness of the cases** | **Selection of controls** | **Definition of controls** | **Comparability of cases and controls** | **Ascertainment of exposure** | **Same method** | **Total**  **Score (/10)** |
| --- | --- | --- | --- | --- | --- | --- | --- | --- |
| Benarroch 2021 | 2 | 1 | 1 | 0 | 1 | 2 | 1 | 8 |
| Bordt 2021 | 2 | 1 | 1 | 0 | 1 | 2 | 1 | 8 |
| Brien 2021 | 2 | 2 | 1 | 1 | 1 | 2 | 1 | 10 |
| Cribiu 2021 | 2 | 0 | 1 | 1 | 1 | 1 | 1 | 7 |
| Dubucs 2022 | 0 | 0 | NA | 1 | NA | 2 | NA | 3 |
| Edlow 2020 | 2 | 1 | 0 | 1 | 0 | 1 | 1 | 5 |
| Garcia-Flores 2022 | 2 | 0 | 1 | 1 | 1 | 1 | 1 | 7 |
| Juttukonda 2021 | 2 | 0 | 1 | 1 | 1 | 2 | 1 | 8 |
| Konstantinidou 2022 | 2 | 1 | 1 | 0 | 1 | 2 | 2 | 9 |
| Kotiloglu-Karaa 2022 | 2 | 1 | 1 | 0 | 0 | 1 | 1 | 6 |
| Laresgoiti-Servitje 2021 | 2 | 1 | 1 | 0 | 0 | 1 | 0 | 5 |
| Mithal 2022 | 1 | 1 | 1 | 1 | 1 | 1 | 0 | 6 |
| Mourad 2021 | 2 | 1 | 0 | 0 | 1 | 0 | 1 | 5 |
| Nizyaeva 2021 | 2 | 1 | 1 | 0 | 0 | 1 | 1 | 6 |
| Rebutini 2021 | 2 | 0 | 1 | 0 | 1 | 2 | 1 | 7 |
| Redline 2022 | 2 | 1 | 1 | 1 | 0 | 1 | 1 | 7 |
| Rolfo 2022 | 2 | 2 | 1 | 1 | 1 | 1 | 1 | 9 |
| Shchegolev 2021 | 2 | 0 | 0 | 0 | 0 | 1 | 1 | 4 |
| Shchegolev 2021 | 0 | 0 | 0 | 1 | 0 | 1 | 1 | 3 |
| Sherer 2021 | 2 | 1 | 1 | 0 | 0 | 1 | 1 | 6 |
| Sureshchandra 2021 | 2 | 0 | 0 | 0 | 0 | 1 | 1 | 4 |
| Taglauer 2021 | 2 | 1 | 1 | 1 | 1 | 1 | 1 | 8 |
| Vargas-Hernandez 2021 | 2 | 1 | 1 | 0 | 1 | 1 | 1 | 7 |
| Verma 2021 | 2 | 0 | 1 | 0 | 1 | 1 | 1 | 6 |
| Watkins 2021 | 2 | 2 | NA | NA | NA | 2 | NA | 6 |
| Wu 2021 | 2 | 0 | 0 | 1 | 0 | 1 | 1 | 4 |
| Zaigham 2022 | 2 | 0 | NA | 1 | NA | 2 | NA | 5 |

Methodological quality assessment of included cohort studies

| **Study** | **Representativeness of the cohort** | **Ascertainment of exposure** | **Assessment of outcome** | **Adequacy of follow up** | **Total Score (/7)** |
| --- | --- | --- | --- | --- | --- |
| Adhikari 2020 | 2 | 2 | 2 | 1 | 7 |
| Argueta 2022 | 2 | 2 | 2 | 0 | 6 |
| Bouachba 2021 | 2 | 2 | 2 | 1 | 7 |
| Boyraz 2022 | 1 | 1 | 2 | 0 | 5 |
| Bunnell 2021 | 2 | 2 | 1 | 1 | 6 |
| Celik 2022 | 2 | 2 | 2 | 1 | 7 |
| Flores-Pliego 2021 | 1 | 2 | 2 | 0 | 5 |
| Garrido-Pontnou 2021 | 0 | 1 | 0 | NA | 1 |
| Glynn 2022 | 2 | 1 | 2 | 0 | 5 |
| Husen 2021 | 2 | 2 | 2 | NA | 6 |
| Ikhtiyarova 2021 | 1 | 1 | 1 | 1 | 4 |
| Jaiswal 2021 | 2 | 2 | 2 | 0 | 6 |
| Jang 2021 | 2 | 2 | 1 | 1 | 6 |
| Lesseur 2022 | 2 | 2 | 2 | 0 | 6 |
| Liu 2021 | 2 | 2 | 2 | 0 | 6 |
| Mandò 2021 | 1 | 2 | 2 | 0 | 5 |
| Meyer 2021 | 2 | 1 | 2 | 0 | 5 |
| Moresi 2021 | 2 | 2 | 2 | 1 | 7 |
| Nadal 2021 | 2 | 2 | 2 | 0 | 6 |
| Peter 2022 | 2 | 2 | 2 | NA | 6 |
| Radan 2021 | 2 | 2 | 1 | NA | 5 |
| Saulle 2021 | 2 | 2 | 2 | 0 | 6 |
| Schwartz 2022 | 1 | 2 | 2 | 1 | 6 |
| Shanes 2020 | 2 | 2 | 2 | 0 | 6 |
| Sotiriou 2022 | 2 | 1 | 2 | 1 | 6 |
| Stenton 2022 | 1 | 2 | 2 | 1 | 6 |
| Ward 2022 | 2 | 2 | 2 | NA | 6 |
| Zhang 2021 | 2 | 2 | 2 | 1 | 7 |
| Zhao, S. 2021 | 2 | 2 | 2 | 0 | 6 |
| Zhao, Y. 2021 | 0 | 2 | 2 | 1 | 5 |
