# Supplementary material for: Potential molecular and cellular mechanisms for adverse placental outcomes in pregnancies complicated by SARS-CoV-2 infection—A scoping review
Source: PLoS One. 2023 Mar 23;18(3):e0283453. doi: 10.1371/journal.pone.0283453 (PMC10035918; doi:10.1371/journal.pone.0283453)
Supplement: S3 File — (DOCX) [file pone.0283453.s003.docx]

**ST3 File. Data Extraction of all studies**

Data extraction of studies included in scoping review

| **Author** | **Ref** | **Journal** | **Year** | **Country** | **Study Design** | **Participant: Placenta**  **(Positive/ Negative)** | **Patient Features** | **Setting** | **Gestational Weeks with COVID-19** | **Testing Method for COVID-19** | **COVID-19 Strain** | **Severity** | **Control** | **Type of Placental Outcome**  **Assessment** | **Method of Measurement** | **Analysis** | **Sig** | **Results** |
| --- | --- | --- | --- | --- | --- | --- | --- | --- | --- | --- | --- | --- | --- | --- | --- | --- | --- | --- |
| Argueta | 19 | iScience | 2022 | USA | Cohort | n=2+ve  n=1-ve | Age  (16-51) | Pregnancy cohort | nr | PCR | Alpha | nr | yes | Molecular  Histo-pathology | RNA-Sequence  Differential expression analysis  Cellular decon-volution | Heat maps | yes | Differences – Placental syncytiotrophoblast target for SARS-CoV-2 infection of pregnant females at term, higher expression of inflammation genes in positive placentas  SARS-CoV-2 infection of placental tissue impacts cellular identity and tissue integrity |
| Benarroch | 20 | Clinical therapeutics | 2021 | USA | Case-control | n=15+ve  n=10-ve | Age  (mean 30)  Gestation age at delivery age (39 weeks) | Hospital | 3^rd^ Trim | PCR | nr | nr | yes | Molecular | Microscopy  Gross exam | Independent Sample t-tests | yes | Differences – Rab5 fluorescence intensity small sig increase (expression found throughout villous syncytiotrophoblast cells and underlying cytotrophoblast cell layers), Rab7 fluorescence intensity large sig increase |
| Bordt | 21 | Science Trans-lational Medicine | 2021 | USA | Case-control | n= 38+ve  n=30-ve | Age  (median 33)  BMI  (29% > 30)  Hispanic (50%)  Most white or ‘other’ | Hospital | 3^rd^ Trim (median 38.7 weeks) | PCR | nr | Asymptomatic (42%)  Mild or mod (50%)  Severe/ Critical (8%) | yes | Molecular | RT-qPCR  Thermo Fisher Scientific  Procart-aplex Analysis | Two-way ANOVA | yes | Differences – Increase placental Fc receptor abundance, IL-10, interferon-stimulated genes, CXCL10, proteins, and density of CD163 Hofbauer cells in male fetus |
| Chen | 22 | Cell Prolifer-ation | 2022 | China | Case study | n=1+ve | Age (30)  Gravida 3, pervious parity 2 | Hospital | 2^nd^ Trim | PCR | Alpha | Severe (100%) | yes | Histo-pathology  Molecular | RNAseq  In situ  Immuno-histochemistry | T-test  Differential Expression Analysis | yes | Differences – Impaired trophoblasts with lower expression of syncytio-trophoblast-related genes, activation of antiviral and inflammatory CD8 T-cells, increased inflammatory responses with complement overactive in macrophages, increased inflammatory factors in placenta |
| Cribiu | 23 | Journal of Clinical Investigation | 2021 | Italy | Case-control | n= 21+ve  n=16-ve | Age (24-38yrs)  Most Caucasian | Pregnancy Cohort | 3^rd^ Trim | PCR | Alpha | nr | yes | Histo-pathology  Molecular | In situ  Nanostring gene expression panel | nr | no | No Differences – Placental histopathology gene expression did not differ between positive or negative women, with exception of high viral load |
| Di Girolamo | 11 | American journal of obstetrics and gynecology maternal-fetal medicine | 2021 | USA, Italy, SwitzerlandChina, UK, Ireland, NetherlandsTurkey, Belgium, Brazil, Spain, Mexico, France | Meta-analysis and system-matic review | n=1008+ve | nr | Multiple settings | nr | PCR | nr | Asymptomatic  Symptomatic | yes | Histo-pathology | (Per study) H&E staining, transmission electron microscopy, FISH, gross exam, immune-histochemistry | Meta-analyses  Odds ratios  Random-effects model  Egger test  Funnel plot | yes | Differences – Women with SARS-CoV-2 infection in pregnancy had a higher risk of FVM (OR, 1.9; 95% CI, 1.3–2.6; P=.002), CIP (OR, 1.94; 95% CI, 1.3–2.8; P=.003), increased perivillous fibrin (OR, 6.8; 95% CI, 2.7–17.0; P<.001), intervillous thrombosis (OR, 3.2; 95% CI, 2.0–5.2; P<.001), and other histopathologic anomalies (OR, 2.0; 95% CI, 1.4–2.7), whereas there was no difference in the risk of developing histopathologic signs of MVM (P=.198) |
| Flores- Pilego | 24 | Cells | 2021 | Mexico | Cohort | n=11+ve  n=4-ve | nr | Hospital | nr | PCR | nr | Mild (45.5%)  Severe (54.5%) | yes | Molecular | Immuno-fluorescence | One-way ANOVA  Plus Normality of D’Agostino  Equal variance  Kruskal-Wallis  Multiple comparison test | yes | Severe cases have higher number of Hofbauer cells, increased vWf expression and decreased Claudin-5 and VE-cadherin in endothelium of decidua and chorionic villi. Extensive histological damage |
| Gabanella | 25 | International Journal of Molecular Sciences | 2022 | Italy | Pilot Study | n=2+ve | Age  (30 or 36) | Hospital | 3^rd^ Trim | nr | nr | nr | yes | Molecular | Padlock Assay immune fluorescence | nr | nr | Overlap of SARS-CoV-2 RNA and mitochondria trophoblastic cells |
| Garcia- Flores | 26 | Nature Commun-cations | 2022 | USA | Case-control | n=12+ve  n=11-ve | Age (21-31.5)  Mostly African American | Pregnancy Cohort | nr | PCR | nr | Asymptomatic (66.7%)  Mild (8.3%)  Severe (25%) | yes | Molecular | RNASeq  rRNA microbiology sequencing | Likelihood ratio test  DE Analysis | no | No changes to microbiology – SARS-CoV-2 infection during pregnancy does not compromise sterility of placenta |
| Hessami | 15 | American Journal of Perinatology | 2022 | USA, Italy, Spain, India, Brazil | Meta-analysis and system-matic review | n=699+ve  n=18326-ve | nr | Between 2020-April 2021 | 3^rd^ Trim | PCR | nr | Asymptomatic  Symptomatic | yes | Histo-pathology | Gross exam  Microscopic exam | Odds ratio with 95% confidence interval  I-Squared tests  Random-effects model | no | No Differences – Maternal inflammatory responses no significance between positive or negative and severity of infection  MVM and FVM comparable but not significant, peri villous fibrin deposition higher in positive placentas, no association of villitis, intervillositis, or deciduitis with infection |
| Juttukonda | 27 | Journal of Reproductive Immunology | 2022 | USA | Case-control | n=16+ve  n=8-ve | Age (mean 30)  Most Hispanic/ Latino | Hospital | 2^nd^ Trim  3^rd^ Trim | PCR | nr | Mild/ Moderate (94%)  Hospitalized (6%) | yes | Histo-pathology  Molecular | PCR  Histology  Immuno-histochemistry | T-Test  ANOVA | yes | Differences –  Decidua in infected patients had higher macrophage and NK count in 3rd trim, T cells were higher in 2nd and 3rd trim, downregulation of IL-6, IL8, IL10, TNFa in 2nd trimester with infection |
| Lesseur | 28 | medRxiv | 2022 | USA | Cohort | n=15+ve  n=30-ve | Age (mean 34)  BMI (mean 25.4)  GA (mean 34.9 weeks)  Most Caucasians, few Black or Hispanic.  Multi/nulli parous (44.4%/55.6%)  (Asthma -17.8%, gest diabetes 22.2%, gest HT 13.3%, chronic HT 8.9%) | Pregnancy  Cohort | nr | SARS-CoV-2 IgG Test | nr | nr | yes | Histo-pathology  Molecular | Histo-pathological exam  Linear models | Fisher’s Exact Test  Wilcoxon Signed Rank  Kruskal-Wallis Test  Spearman correlation  Linear regression models | yes | Differences – Decrease 2 trophoblast genes (PSG3, CBG3), increase 3 immune genes (CXCL10, TLR3, DDX58), higher BMI lead to more IgG seropositive participants, Hispanic or non-Hispanic black had increased infectivity  No Differences – Placental weight, GA at delivery, other maternal characteristics with age and medical conditions, histopathology |
| Liu | 29 | Frontiers in Pediatrics | 2021 | China | Cohort | n=31+ve  n=49-ve | Age (30/5+/-3.4)  BMI (29.93+/-0.38)  Gravidity/ Parity (2/0)  Conditions | Pregnancy  Cohort | nr | PCR | nr | Moderate (100%) | yes | Histo-pathology  Molecular | H&E Staining  ELISA | ANOVA  Student T-Test  Mann-Whitney Test  Chi Square  Linear mixed effect models  Bonferroni correction | yes | Differences – Elevated myocardial enzymes, serum lactate dehydrogenase, IL-2, IL-6, interferon gamma, TNF-a, and mtDNA placental levels; abnormal mitochondria activity, hypoxia and ischemia and MVM present in positive infected placentas |
| Mandò | 30 | Antioxidants | 2021 | Italy | Cohort | n=30+ve  n=16-ve | Age (Asymp 23.8+/-5.1, symp 25.4+/-4.5)  BMI (Asymp 13.2+/-5.3, symp 11.1+/-4.1)  Caucasian (Asymp 80%, symp 63%)  GA (Asymp 39.1+/-1.4, symp 38.9+/-1.4) | Pregnancy  Cohort | 3^rd^ Trim | PCR | nr | Asymptomatic (40%)  Symptomatic (60%) 🡪 Severe (16.7%) | yes | Molecular | mtDNA levels  DNA oxidative damage  Gene expression | Kolmogorov-Smirnov test  ANOVA  Kruskal-Wallis test  Mann-Whitney Test  Chi Square | yes | Differences – Higher DNA oxidative damage levels with lower placental mtDNA levels, decrease oxidative defence gene expression (CAT, GSS, GSR) and respiratory chain subunits (NDUF49, SDHA, COX411) and mitochondrial dynamics of fusion and fission (DNM1L, FIS1) in comparison to controls  No Differences – Gestational age, BMI, maternal age no significance |
| Mourad | 31 | Nature: Scientific Reports | 2021 | USA | Case-control | n=66+ve  n=18-ve | Age (mild/severe 28.6/36.0)  BMI (mean 30)  GA (mild/severe 39/36.3)  Hispanic (73%) | Pregnancy Cohort | 3^rd^ Trim  (Asymp/ mild 38 weeks; severe 35.9 weeks) | PCR | nr | Asymptomatic/mild (89.4%)  Severe (10.6%) | yes | Histo-pathology  Molecular | PCR  Immuno-fluorsence | Fisher’s Exact Test  Welch’s T-Test  Wilcoxon-Mann-Whitney test  Krukal-Wallis  Post hoc Tukey test | yes | Differences – ACE2 levels and furin lower in asymp/ mild compared to severe, placental mRNA of IFITM1 and IFITM3 upregulated  No Differences – Between asymptomatic/mild and severe disease groups with respect to the frequency of MVM and FVM lesions, acute inflammatory processes, chronic inflammatory processes, or lesions, TMPRSS2 levels same in asymp/mild compared to severe, expression of SARS-CoV-2 entry factors and IFITM transcripts and placental histopathology |
| Nizyaeva | 32 | Bulletin of Russian State Medical University | 2021 | Russia | Case-control | n=66+ve  n=40-ve | Age (mean 30)  BMI (mean 27.1) | Pregnancy Cohort | 3^rd^ Trim | PCR | nr | Asymptomatic (22.7%)  Mild (38.0%)  Mod (30.2%)  Severe (9.1%)  Death (1.5%) | yes | Histo-pathology  Molecular | H&E Staining  Micro and macroscopic exam  Immuno-histo-chemistry | nr | yes | Differences – Higher rate of villous infarction in positive infection, elevated TNF-a and IL-8, downregulation of IL4 |
| Redline | 33 | American Journal of Surgical Pathology | 2022 | USA | Case-control | n=271+ve  n=8006-ve | nr | Hospital | nr | PCR | nr | Most mild/mod  Severe (10%)  (From 111/271) | yes | Histo-pathology  Molecular | Immuno-histo-chemistry | Chi Square | yes | Differences – Diffuse SARS-CoV-2 placentitis more common in preterm births |
| Rolfo | 34 | Bio  medicines | 2022 | Italy | Case-control | n=41+ve  n=12-ve | Age (33)  GA (38 weeks) | Hospital | nr | PCR | nr | Mild/mod | yes | Histo-pathology  Molecular | Transmission electron microscopy  PCR  Enzymatic activity assay  Lipid peroxidation measurement | Mann-Whitney U Test | yes | Differences – TBARS (lipid peroxidation marker), HIF-1a, CAT and SOD catalase and superoxide dismutase transcript and enzymatic levels higher in covid positive  No Differences – Placental weight in comparison to control group |
| Saulle | 35 | Cells | 2021 | Italy | Cohort | n=15+ve  n=6-ve | Age (Positive 32, negative 33.5)  BMI (23.4) | Hospital | 3^rd^ Trim | nr | nr | Asymptomatic | yes | Molecular | miRNA profiling  RNA extraction  Multiplex ELISA  Ogene  Placental biopsy collection | Student T-Test  Mann-Whitney U test  Multivariate linear regression model | yes | Differences - miRNA expression in placental biopsies increased (miR-29a-29c-21-98), sig increase of host antiviral effector genes in placenta (miR-146,150,155) with slight increase of activation markers, chemokines, pro-inflammatory, anti-inflammatory cytokines  No Differences – BMI same |
| Shchegolev | 36 | Bulletin of Experimental Biology and Medicine | 2021 | Russia | Case-control | n=23+ve  n=7-ve | Age (22-48) | nr | nr | nr | nr | Mild or mod | yes | Histo-pathology  Molecular | Imuno-histochemistry | nr | yes | Differences – Increased syncytial knots and VEGF expression in placenta with positive infection 🡪 Higher number with higher severity of infection |
| Sherer | 37 | American journal of obstetrics and gynecology | 2021 | USA | Case-control | n=22+ve  n=11-ve | Age (med 29)  Asian (6.1%)  Black (18.2%)  Caucasian (33.33%)  Other (42.4%) | Pregnancy Cohort | nr | PCR | nr | Mild or mod | yes | Molecular | PCR  Western blot | Exact Wilcoxon 2-Sample Tests | no | No Differences – IL1beta or IL6 mRNA expression no significant differences between infected and non-infected patients |
| Suhren | 16 | Placenta | 2022 | nr | Systematic review and meta analysis | n=1452+ve | nr | nr | nr | nr | nr | nr | no | Histo-pathology | Meta-analysis | nr | nr | Did not reveal any COVID-19-specific placenta changes. The incidence of both vascular and inflammatory lesions was mainly comparable to that of non-COVID-19 pregnancies |
| Suresh-chandra | 38 | bioRxiv (preprint) | 2021 | USA | Case-control | n=13+ve  n=19-ve | Age (mean 32)  BMI (mean pre-pregnancy 24.1 | nr | 1^st^ Trim (7.7%)  2^nd^ Trim (15.4%)  3^rd^ Trim (76.9%) | PCR | nr | Asymptomatic  Mild | yes | Molecular | Multispectral flow  Cytometry  Single cell RNA sequencing | Unpaired T-Test  Welch’s Correction  One-way ANOVA  Holm Sidak’s Multiple comparisons test | yes | Differences – Positive infection associated with loss of decidual macrophages, attenuation of type 1 interferon signalling, upregulation of inflammatory chemokines and cytokines in macrophages, remodelling T-cell compartment with increased CD69+, decreased regulatory T-cells, CD8 effector memory T-cells expanded into decidua, type 1 IFN signalling increased in decidual T-cells but not macrophages, T-cell receptor diversity reduced in decidual |
| Taglauer | 39 | American Journal of Pathology | 2021 | USA | Case-control | n=16+ve  n=8-ve | Age (mean 30)  Mostly Caucasian | Pregnancy Cohort | 2^nd^ Trim  Or  3^rd^ Trim | PCR | nr | Mild or moderate (100%) | yes | Molecular | Immunohisto-chemistry  ELISA  PCR | One-way ANOVA  Tukey post hoc analysis  Two-way ANOVA | yes | Differences – 3^rd^ trim decreased ACE2 expression and upregulated ACE2 mRNA compared to 2^nd^ trim and controls, 3rd trim increased ADAM17 activity compared to controls  No Differences – 2^nd^ trim similar placental ACE2 expression as controls |
| Verma | 40 | Med/ Cell Press | 2021 | USA | Case-control | n=5+ve  n=5-ve | Age (17-32)  Caucasian (25%)  Black (75%) | Hospital | 2^nd^ Trim (25%)  3^rd^ Trim (75%) | PCR | nr | Asymptomatic or mild | yes | Histo-pathology  Molecular | H&E Staining  Immunohisto-chemistry  Western blot | One-way ANOVA | yes | Differences – Preterm placentas showed trophoblast giant cells in decidua and markers of inflammation in fetal membranes and umbilical cord, significant reduction of ACE2 expression after infection  No Differences – Placentas infected at term showed no significant histopathological changes, no histological correlation between symptomatic and asymptomatic placentas infected |
| Wu | 41 | Cell Proliferation | 2021 | China | Case-control | n=11+ve  n=4-ve | Age (25-34) | Pregnancy Cohort | 3^rd^ Trim (22-31 weeks) | PCR  / IgG Test | Alpha | Mild or mod | yes | Molecular | Immuno-staining  Luminex assay | Paired Sample T-Test | yes | Differences – CD14+, IP-10, IFN-y higher in infected placentas |
| Zhao | 42 | Frontiers in Immunology | 2021 | China | Cohort | n=33+ve  n=8-ve | Age (28-43) | Hospital | nr | nr | nr | Mild or moderate | yes | Histo-pathology  Molecular | Gross exam  Multiparameter flow cytometry  Luminex assay  Histological analysis  Immunohisto-chemistry  Immuno-fluorescence  PCR | ANOVA | yes | Differences – Mild hypoxic damages, increased CD68+ macrophages infiltration, ACE2 levels highest in first trimester, then gradually decreased to the lowest amount in third trimester  No Differences – No TMPRSS2 detected in early, middle, late pregnancy by immunofluorescence and not co-expressed with ACE2 in all stages |

nr = not recorded; GA = gestational age; PCR = polymerase chain reaction; MVM = maternal vascular malperfusion; FVM = fetal vascular malperfusion; BMI = body mass index; HT=hypertension; TNFa = tumor necrosis factor alpha; IFNy = interferon gamma; H&E Staining = hematoxylin and eosin staining; ELISA = enzyme linked immunosorbent assay; FISH = fluorescence in situ hybridization; RT-qPCR = reverse transcription quantitative polymerase chain reaction
