# Supplementary material for: Potential molecular and cellular mechanisms for adverse placental outcomes in pregnancies complicated by SARS-CoV-2 infection—A scoping review
Source: PLoS One. 2023 Mar 23;18(3):e0283453. doi: 10.1371/journal.pone.0283453 (PMC10035918; doi:10.1371/journal.pone.0283453)
Supplement: S4 File — (DOCX) [file pone.0283453.s004.docx]

**ST4 File. Acronyms and abbreviations.**

| Acronym | Definition |
| --- | --- |
| ACE | Angiotensin converting enzyme |
| ACE2 | Angiotensin converting enzyme two receptor protein |
| ADAM17 | A disintegrin and metalloprotease domain 17 |
| AT1 | Angiotensin II receptor 1 |
| CAT | Catalase |
| CCL3 | C-C motif chemokine ligand 3 |
| CCL4 | C-C motif chemokine ligand 4 |
| CCL5 | C-C motif chemokine ligand 5 |
| CD8 | Cluster of differentiation 8 |
| CD14 | Cluster of differentiation 14 |
| CGB3 | Chorionic gonadotropin subunit beta 3 |
| CH25H | Cholesterol 25-hydroxylase |
| COX4I1 | Cytochrome C oxidase subunit 4I1 |
| CXCL10 | C-X-C motif chemokine ligand 10 |
| DDX58 | DExD/H-Box helicase 58 |
| DNM1L | Dynamin-1-like protein |
| FIS1 | Mitochondrial fission 1 protein |
| GPCR | G-protein coupled receptor |
| GSS | Glutathione synthetase |
| HIF-1a | Hypoxia inducible factor 1 alpha |
| IL1B | Interleukin 1 beta |
| IFI6 | Interferon alpha-inducible protein 6 |
| IFITM1 | Interferon induced transmembrane protein 1 |
| IFITM3 | Interferon induced transmembrane protein 3 |
| IFNa | Interferon alpha |
| IFNB | Interferon beta |
| IFNy | Interferon gamma |
| IL-4 | Interleukin-4 |
| IL-6 | Interleukin-6 |
| IL-8 | Interleukin-8 |
| IL-10 | Interleukin-10 |
| IP-10 | Interferon gamma-induced protein 10 |
| MIF | Macrophage migration inhibitory factor |
| MIG | Monokine induced by interferon-gamma |
| mtDNA | Mitochondria DNA |
| MX1 | MX dynamin like GTPase 1 |
| NK | Natural killer cells |
| NDUFA9 | NADH:Ubiquinone oxidoreductase subunit A9 |
| OAS1 | Oligoadenylate synthetase protein 1 |
| PSG3 | Pregnancy specific beta-1-glycoprotein 3 |
| RAB5 | Ras-related in brain 5 |
| RAB7 | Ras-related in brain 7 |
| RAS | Renin angiotensin system |
| TBARS | Thiobarbituric acid |
| TLR3 | Toll-like receptor 3 |
| TMPRSS2 | Transmembrane protease serine 2 |
| TNFa | Tumor necrosis factor alpha |
| sACE2 | Soluble angiotensin converting enzyme two receptor protein |
| scRNAseq | Single cell RNA sequencing |
| SDHA | Succinate dehydrogenase complex flavoprotein subunit A |
| SOD | Superoxide dismutase |
| VEGF | Vascular endothelial growth factor |
